# Poly(amidoamine) Dendrimers as Nanocarriers for 5-Fluorouracil: Effectiveness of Complex Formation and Cytotoxicity Studies

**DOI:** 10.3390/ijms222011167

**Published:** 2021-10-16

**Authors:** Magdalena Szota, Katarzyna Reczyńska-Kolman, Elżbieta Pamuła, Olga Michel, Julita Kulbacka, Barbara Jachimska

**Affiliations:** 1 Jerzy Haber Institute of Catalysis and Surface Chemistry Polish Academy of Sciences, 30-239 Krakow, Poland; magdalena.szota@ikifp.edu.pl; 2 Department of Biomaterials and Composites, Faculty of Materials Science and Ceramics, AGH-University of Science and Technology, 30-059 Krakow, Poland; kmr@agh.edu.pl (K.R.-K.); epamula@agh.edu.pl (E.P.); 3 Department of Molecular and Cellular Biology, Faculty of Pharmacy, Wroclaw Medical University, 50-367 Wroclaw, Poland; michel.olga.maria@gmail.com (O.M.); julita.kulbacka@umw.edu.pl (J.K.)

**Keywords:** PAMAM dendrimers, 5-fluorouracil, PAMAM–5FU complexes, controlled drug delivery systems, dendrimer cytotoxicity, nanomedicine, nanomaterials

## Abstract

Two generations of positively charged poly(amidoamine) dendrimers (PAMAMs) were selected for study as potential carriers for the anticancer drug 5-fluorouracil (5FU), a drug primarily used in the treatment of colorectal cancer. Analytical techniques, such as UV-Vis spectrophotometry, NMR Spectroscopy and Laser Doppler Velocimetry (LDV), have shown that the most critical factor determining the formation of a PAMAM–5FU complex is the starting components’ protonation degree. The tests confirmed the system’s ability to attach about 20 5FU molecules per one dendrimer molecule for the G4PAMAM dendrimer and about 25 molecules for the G6PAMAM dendrimer, which gives a system yield of 16% for the fourth generation and 5% for sixth generation dendrimers. Additionally, using the QCM-D method, the adsorption efficiency and the number of drug molecules immobilized in the dendrimer structure were determined. A new aspect in our study was the determination of the change in zeta potential (ζ) induced by the immobilization of 5FU molecules on the dendrimer’s outer shell and the importance of this effect in the direct contact of the carrier with cells. Cytotoxicity tests (resazurin reduction and MTS tests) showed no toxicity of dendrimers against mouse fibroblast cells (L929) and a significant decrease in cell viability in the case of four human malignant cell lines: malignant melanoma (A375), glioblastoma (SNB-19), prostate cancer (Du-145) and colon adenocarcinoma (HT-29) during incubation with PAMAM–5FU complexes. The purpose of our work was to investigate the correlation between the physicochemical properties of the carrier and active substance and the system efficiency and optimizing conditions for the formation of an efficient system based on PAMAM dendrimers as nanocarriers for 5-fluorouracil. An additional aspect was to identify potential application properties of the complexes, as demonstrated by cytotoxicity tests.

## 1. Introduction

Cancer is one of the most common causes of death worldwide [1]. Currently, chemotherapy, which uses chemicals to block the growth or kill rapidly growing cancer cells, thereby also killing healthy cells, plays a major role in cancer treatment. Due to chemotherapy’s highly negative impact on the human body, alternative methods of treatment for neoplastic diseases are sought. A promising form of therapy may be the implementation of a Controlled Drug Delivery System (CDDS) [2]. The development of a CDDS requires interdisciplinary knowledge, including chemistry, pharmaceutical sciences, medical sciences, or materials engineering [3]. CDDS’ task is to eliminate the problems that arise during conventional drug administration by extending the active ingredient’s release time, increasing the solubility, or stopping the process of accumulation of the active substance in healthy body tissues by releasing it directly to target cells [2,3]. Nanotechnology has played an essential role in medicine for several decades, including cancer diagnostics and treatment [4]. Nanomedicine involves the application of nanodimensional materials characterized by size ranging from 1–100 nm. Nanoparticles, due to their size, have unique physicochemical, mechanical, electrical, and biological properties and may find a wide range of applications in nanobiotechnology, tissue engineering, and as sensors, imaging agents, or drug carriers. Despite their excellent properties, currently only 49 nanoparticle-based products have been approved by the FDA for clinical practice, of which only 7 are used in anticancer therapy [5]. Nanomedicine is playing an increasingly important role in pharmaceutical development, but regarding nanoparticles, many challenges need to be considered during manufacturing, such as regulations related to the approval of a nanoproduct for clinical use. In particular, there are a number of nanoparticle properties to consider, such as physicochemical characterization, assessment of biocompatibility, pharmacokinetics and pharmacodynamics. Additionally, nanocarrier-based formulations must also prove the biocompatibility of each component in the formulation, such as in vivo aggregation, controlled drug release, recognition by the reticuloendothelial system, and response to immune activation. This whole process of developing and implementing an effective nanoproduct is long and costly, resulting in such limited results in clinical practice [6]. The most studied nanoparticles are liposomes, polymer nanoparticles, micelles, carbon nanotubes, and dendrimers [7]. Poly(amidoamine) dendrimers (PAMAMs) are among the most widely used synthetic nanoparticles in controlled drug delivery systems [8,9]. They are characterized by monodispersed, spherical shapes and a defined, branched structure containing strictly defined types of functional groups [9]. The structure of dendrimers consists of three main architectural elements: (i) core, (ii) branches, and (iii) surface functional groups attached to the branches. This characteristic dendrimer structure creates internal cavities within the structure, allowing drug encapsulation [10]. The presence of functional groups allows the active agent molecules to be immobilized on the carrier surface [7]. Access to the interior of a molecule for the active agent strictly depends surrounding environment, especially on the degree of protonation of the external functional groups [7,8].

There are two ways to synthesize dendrimers—backward synthesis and convergent synthesis. In the first case, dendrons are synthesized first and then attached to the core. The second type consists of coating the core with successive layers of mers that form the appropriate dendrons and ultimately the entire dendrimer. Each subsequent layer around the core is called a generation. There are functional groups on the surface of the dendrimer, the number of which increases twice with each generation [11,12]. Cationic PAMAM dendrimers have an ethylenediamine core from which, depending on the generation of a dendrimer, a certain number of branches terminated with amino-NH_2_ groups depart. The physicochemical properties of dendrimers rely not only on external branching units but also on terminal groups. They determine the behavior of dendrimers in a given environment, the ability of ligands to bind, and cytotoxicity [11]. Despite their unique properties, dendrimers have not yet been implemented into medical practice on a larger scale. The first marketed product based on dendrimers is Vivagel^®^, an antiviral and antibacterial agent developed by pharmaceutical company Starpharma Ltd. [13]. The implementation of a dendrimer-based product into clinical practice proves their practical applicability.

5-fluorouracil (5FU) is an anticancer drug used mainly in the treatment of colorectal cancer, other cancers of the gastrointestinal tract, and breast cancer [14]. Chemically, 5FU is 5-fluoropyrimidine-2,4-dione, a uracil derivative containing a fluorine atom on the fifth carbon (C-5) [15]. The form that 5FU takes in the human body environment (neutral or ionized) plays a significant role in the migration of molecules through cell membranes and interaction with proteins or carriers [16]. From a theoretical point of view, the form that 5FU takes strictly depends on the solution’s pH. 5FU in aqueous solutions occurs in a neutral form below pH 8 and a deprotonated form at alkaline pH (>8). The negative charge is mainly located on nitrogen atoms N1 (63%) and N3 (37%) [16,17,18].

To maintain the therapeutic level of 5FU in serum, a specific dose of the active agent would have to be administered continuously, increasing the drug’s concentration up to values that evoke toxicity. Additionally, the response rate of advanced colorectal cancer to pure 5FU is only 10%, but when combined with other active substances, this response rate increases to 45% [15]. The delivery of 5FU to the target site using a non-cytotoxic vehicle would improve the therapy’s effectiveness by improving the response to treatment and, at the same time, reducing side effects.

The study’s main aim was to investigate the correlation between the physicochemical properties of the carrier and the active substance and the efficiency of the PAMAM–5FU complex formation. In this context, an important aspect was to define the mode of interaction between the carrier molecules and 5FU. Therefore, several analytical methods were used in the research, such as UV-Vis spectroscopy, dynamic light scattering (DLS), quartz microbalance with monitored energy scattering (QCM-D) and nuclear magnetic resonance spectroscopy (NMR). Potentially, 5FU molecules, mainly through electrostatic interaction, can be immobilized on the outer shell of the dendrimer or through hydrophobic interactions inside the carrier. Furthermore, changes in the molecule’s charge confirm the presence of 5FU molecules on the outer shell. On the other hand, NMR tests indicate the presence of the active agent in both locations. An essential parameter of the system influencing the formation of the active complex in the case of both studied generations of PAMAM dendrimers is the degree of protonation of the molecule. Moreover, cytotoxicity tests confirmed the lack of toxicity of the used carrier, regardless of the generation used. The studies of the selectivity of the action of the complexes concerning a few selected tumor cell lines showed a positive effect of the immobilization of the active agent in the structure of the dendrimer.

## 2. Results and Discussion

### 2.1. Physicochemical Properties

**Figure 1:**
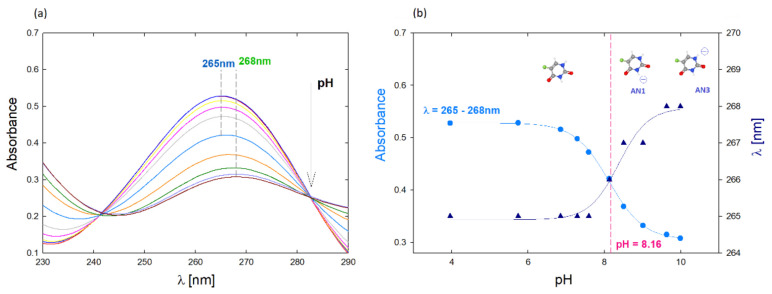
(**a**) UV-Vis spectrum of the aqueous 5-FU solution depending on the pH, c = 7.7 µM; (**b**) Effect of pH on the position of the maximum absorbance of the spectrum and the dependence of absorbance for λ = 265 nm on the pH of the solution.

The structure of 5FU is characterized by the presence of a benzene ring, amino and oxygen groups and a fluorine atom substituted in place of hydrogen at five position [19]. Due to its structure, the 5FU molecule has two potential sites for protonation and deprotonation. According to the literature data, the neutral form of 5FU occurs below pH = 8, while the deprotonated form occurs in alkaline conditions (pH > 8) [16]. The effect of pH on the change of the 5FU spectrum was controlled using UV-Vis spectroscopy for the concentration 7.7 µM (Figure 1a). As the pH increases, the position of the peak maximum in the UV-Vis spectrum changes. In the range of pH 4–7, it is located at the wavelength λ_max_ = 265 nm. The increase in pH causes a gradual shift of the peak position, which for pH 10 is located at the wavelength λ_max_ = 268 nm (Figure 1b). From pH 4 to 8, the change in the maximum absorbance value and the shift of the peak characteristic for 5FU are visible. According to the literature data, under these conditions, 5FU occurs in a neutral form [16].

A significant shift of the peak towards higher wavelengths and a sharp decrease in absorbance is observed at pH above 8, which is probably the result of deprotonation of the N1 and N3 amino groups (Figure 1b). The UV-vis method was also used to quantify the drug in the tested complexes. The calibration curve was determined based on spectra obtained for 5FU solutions in the concentration range c = 7.7–192 µM. Based on the UV-vis spectra, the molar extinction coefficient (ε) was determined for an aqueous 5FU solution depending on the pH (Supplementary Materials, Figure S1) [20]. The molar absorption coefficient decreases with increasing pH. In the case of pH equal to 7.5 it is ε_5FU_ = 6330 M^−1^ cm^−1^, while for pH 10 it is ε_5FU_ = 4515 M^−1^ cm^−1^, which is consistent with the literature data [21]. The value of ε in neutral pH reported in the literature ranges from 7000 M^−1^ cm^−1^ to 6300 M^−1^ cm^−1^ [22,23].

Precise physicochemical characteristics of the carrier molecules are fundamental in the development of functional carrier–drug complexes. A significant advantage of dendrimers in the present context is their defined structure and, at the same time, a precisely defined type and number of functional groups [24]. Functional groups in the dendrimer structure have a key influence on the physicochemical properties that determine the surface charge’s value and potential interactions with ligand molecules or solvent molecules [25]. Dendrimers are branched polyelectrolytes, the properties of which differ from linear polyelectrolytes [25,26]. Using the dynamic light scattering (DLS) method, the diffusion coefficient of the molecules was determined, and on this basis, the hydrodynamic radius of the molecule was determined using the Stokes–Einstein equation [27,28]:

(1) $$R_{H}=\frac{kT}{6\pi \eta D}$$

were *D*—diffusion coefficient *k*—Boltzmann’s constant, *T*—temperature, *η*—viscosity, and *R_H_*—hydrodynamic radius [29].

The hydrodynamic radius of G4PAMAM and G6PAMAM molecules was determined for the concentration of dendrimers of 70 and 17 µM, respectively, at the solution’s ionic strength I = 1 × 10^−2^ M NaCl. The dendrimer hydrodynamic radius value for the lower generation is 2.45 ± 0.06 nm, and for the higher generation is 3.75 ± 0.19 nm. Similar values were obtained for dendrimers using the NMR method, 2.08 ± 0.13 nm for G4PAMAM and 2.95 ± 0.14 nm for G6PAMAM [30].

The protonation degree of dendrimers is a complex process due to the diversity of their structure and the presence of primary and tertiary amines. According to the literature data, the protonation of PAMAM dendrimers of higher generations (above 2) consists of 3 main steps. In the first stage primary amines present on the particle surface are independently protonated with microconstant pK^(I)^ = 9.0, whereas tertiary amines present inside the structure are deprotonated. The second step involves protonation of tertiary amines with a microconstant pK^(II)^ = 5.8, except for the central tertiary amine group. This group protonates in the third step of protonation (pK^(III)^ = 3.5), while this process has no significant impact on the total charge of the dendrimer molecule [31].

Using electrophoretic mobility measurements, we indicated the surface charge of the fourth and generation PAMAM dendrimers and determined the values of the effective charge (*N_c_*) and the degree of ionization (α_e_) of the dendrimers as a function of generation and pH, which corresponded to the conditions of complex formation (pH = 5.5, 7.5, and 10).

The effective charge of a molecule (*N_c_*) is defined as the average number of charges present in one molecule and was calculated using the Stokes–Lorenz equation [22,23,24,25,26,27,28,29,30,31,32]:

(2) $$N_{c}=\frac{6\pi \eta R_{H}}{e}\mu_{e}$$

where *μe* = ⟨U⟩/E and ⟨U⟩ is the average migration velocity of dendrimer molecules in a uniform electric field E and *e* = 1.602 × 10^−19^ C [27].

Based on the effective charge of the molecule we can find the effective ionization degree (α_e_), which is defined as [25]:

α_e_ = *N_c_/N_m_* (3)

where *N_m_* is nominal charge of molecule which equals 64 for G4PAMAM and 256 for G6PAMAM.

The zeta potential was determined for aqueous solutions of both generations of dendrimers as a function of pH. Measurements were carried out in the pH range of 3–11. For lower pH, the zeta potential value was kept at a high level (60–80 mV). In the range of pH 7–8, a rapid decrease in potential is observed, and the dendrimer changes into a negatively charged form in a strongly alkaline environment (pH > 10). The results of the zeta potential measurements correlate with the literature data regarding the degree of protonation of the dendrimers. In general, dendrimers in the pH range from 3.5 to 8 PAMAM are characterized by the highest protonation of surface groups, enhanced by the protonation of the internal groups and the synergy effect of the primary and tertiary amines.

The strong influence on the zeta value of the potential is observed when changing the solution’s ionic strength. The increasing ionic strength results in a sharp decrease in the zeta potential due to the condensation of counterions on the particle surface. Thus, higher generation dendrimers are characterized by a relatively higher potential, which is consistent with the literature data [33,34].

Table 1 presents the zeta potential values (ζ), the effective charge (*N_c_*), and the effective degree of ionization (*α_e_*) depending on the dendrimer generation and the solution pH. The overall relationship between effective charge and pH is similar for both generations, while N_c_ decreases with increasing pH. G6PAMAM particles have a higher effective surface charge than lower generation particles. The literature data show the exact relationship between the production of PAMAM dendrimers and their effective charge [35]. The inverse relationship is observed for the effective degree of ionization, the value of which depends on the number of surface functional groups. The degree of ionization is higher for G4PAMAM, which has four times fewer amino groups than G6PAMAM.

### 2.2. Effectiveness of Complex Formation

Complexes of PAMAM dendrimers with 5-fluorouracil were formed under different conditions considering factors such as carrier/drug molar ratio, pH of complex formation, and dendrimer generation. The research was carried out in a wide range of PAMAM/5FU molar ratios from 1:50, 1:150, 1:250, 1:500 to 1:800. The optimum system was obtained with a high excess of the drug relative to the functional groups present in the dendrimer molecule as 1:800. After dialysis of the obtained complexes, the concentration level of the drug immobilized in the support structure was determined using UV-Vis spectroscopy.

**Figure 2:**
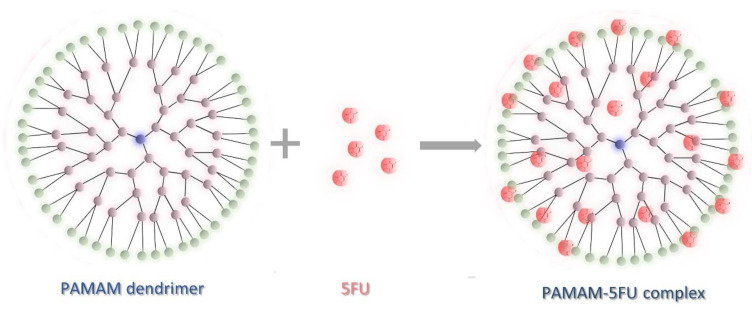
Schematic illustration of PAMAM dendrimer/5-fluorouracil complex formation.

As the starting concentration of 5FU increased, the complex formation efficiency increased. A significant molar excess of 5-fluorouracil plays a crucial role in complex formation. The highest amount of active ingredient was obtained for the PAMAM/5FU molar ratio of 1:800. The dendrimer-5-fluorouracil complex formation process is shown schematically in Figure 2.

**Figure 3:**
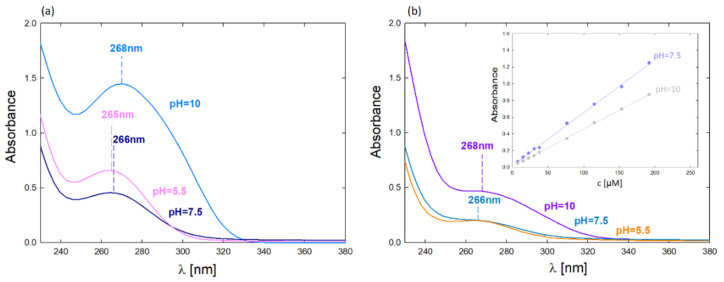
UV-Vis spectra of complexes after dialysis depending on the pH of complex formation (molar ratio 1:800): (**a**) G4PAMAM–5FU (c_PAMAM_ = 17.5 µM); (**b**) G6PAMAM–5FU (c_PAMAM_ = 4.3 µM); (c) calibration curves for 5FU and their dependence of pH.

As mentioned, the complex formation efficiency was monitored using the UV-Vis method. Figure 3 shows the UV-Vis spectra of the formed PAMAM–5FU complexes. The UV-Vis spectra’s maxima are in the wavelength range λ_max_ = 264–268 nm, depending on the pH. The highest absorbance value of the dendrimer generation corresponds to the complex formed at alkaline pH (pH = 10). In G4PAMAM, greater efficiency was obtained with the formation of the complex at pH = 5.5, while in the case of G6PAMAM, the efficiency is slightly higher for the complex formed at pH = 7.5.

The quantitative attachment of 5-fluorouracil molecules is much higher for G4PAMAM, where approximately 20 drug molecules per one dendrimer molecule were bound (Table 1). Buczkowski et al. obtained similar system efficiency indicating 30 ± 4 active sites in G4PAMAM that could bind 5FU [20]. For G6PAMAM, about 25 drug molecules were bound per one dendrimer molecule.

**Figure 4:**
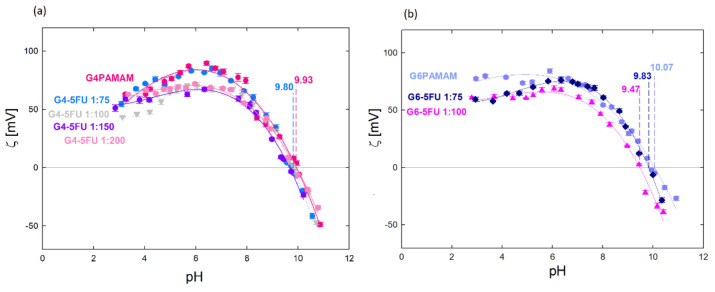
Zeta potential values for: (**a**) aqueous solution of G4PAMAM and G4-5FU complexes for different molar ratios (1:75, 1:100, 1:150, 1:200), c_PAMAM_ = 70 µM; (**b**) an aqueous solution of G6PAMAM and G6-5FU complexes for various molar ratios (1:75, 1:100), c_PAMAM_ = 17 µM.

The effect of 5FU immobilization in the structure of dendrimers was monitored with the use of electrophoretic mobility. Figure 4 shows the zeta potential measurements for the aqueous solutions of G4PAMAM, G6PAMAM, G4-5FU, and G6-5FU. Based on these measurements, the dendrimer–drug molar ratio’s influence on the zeta potential value and the efficiency of 5FU incorporation into the support structure were determined. Increasing the ratio above 1:100 did not change the system’s zeta potential, although the carrier’s highest efficiency was obtained under the conditions of 1:800 (Figure 4a). Complex formation with a significant excess of the drug does not change its effective charge of the dendrimer system but allows for more efficient drug encapsulation.

The higher drug attachment efficiency for the higher dendrimer/5FU molar ratio resulted in the value of the zeta potential of the system (Table 2). The zeta potential is lower for the complex containing the higher amount of incorporated 5FU, while the isoelectric point position is independent of the 5FU concentration and occurs at pH = 9.80. The change in the system’s zeta potential towards a more negative one indicates that the immobilization of the 5FU molecules neutralizes the positive charge from the amino groups present on the surface of the dendrimer molecule. The isoelectric point for the G6-5FU complexes shifts towards lower values when a more active agent is immobilized (iep = 9.83 for the 1:75 complex; iep = 9.47 for the 1:100 complex) (Figure 4b). The zeta potential value of the G4-5FU complex was reduced by about 17 mV in the pH range 3–7, resulting in a charge reduction of 22%. Relating this value to the number of surface primary amines in the G4PAMAM structure, we can estimate that 5-fluorouracil molecules loaded approximately 14 surface groups. In the case of the G6-5FU complex, the zeta potential was reduced by 11% relative to the pure dendrimer, which, in terms of the numerous G6PAMAM surface groups, indicates that approximately 28 amino groups are loaded with 5FU molecules. This may indicate that 5FU will be mainly located on the carrier’s surface in the higher generation. The immobilization of the drug inside the carrier structure may be difficult in this case due to the three-dimensional structure of G6PAMAM and the denser packing of functional groups, which may constitute a barrier to the free penetration of 5FU molecules inside the structure. According to our observations, due to deprotonation localized on nitrogen atoms in the 5FU structure at pH > 8, a process of immobilization of molecules in the carrier structure occurs due to protonation of part of the dendrimer surface groups under these conditions. The significant effect of the protonation degree of both components on the change in zeta potential and the efficiency of complex formation provides an influence of electrostatic interactions on the nature of the interaction between PAMAM dendrimers and 5-fluorouracil.

**Figure 5:**
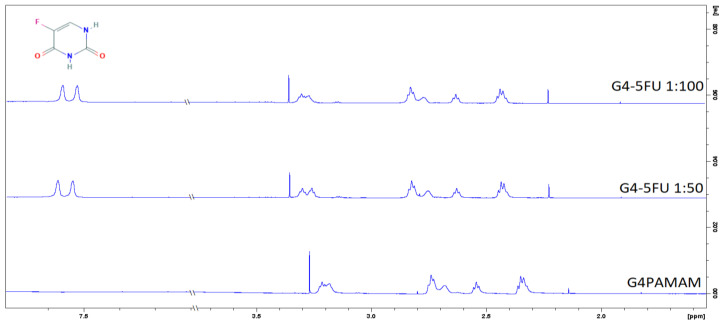
H^1^ NMR spectra for G4PAMAM and G4-5FU complexes in 1:50 and 1:100 molar ratios.

The effect of the 5FU particle immobilization on the dendrimer structure was additionally determined using the NMR method. Figure 5a shows the fourth generation dendrimer spectrum, characterized by several chemical shifts in the range of 2.1–3.2 ppm. According to the literature data, the signal around 2.8 ppm comes from protons of the methylene groups associated with terminal amino groups and internal quaternary amines. The chemical shift around 3.2 ppm from protons located on amino groups in the amide bond [36]. The amide bond is a repeating element in the dendrimer molecule and occurs both inside the structure and near the location of the surface groups. Thus, the visible shift in the NMR spectra shows the effect of 5FU on both internal and surface groups. This means that 5FU exists in both locations, but a strict division between both locations cannot be determined.

In the H^1^ NMR spectra obtained for the complexes, an additional peak is observed around σ = 7.5 ppm, which comes from a proton present in the 5FU structure [37].

**Figure 6:**
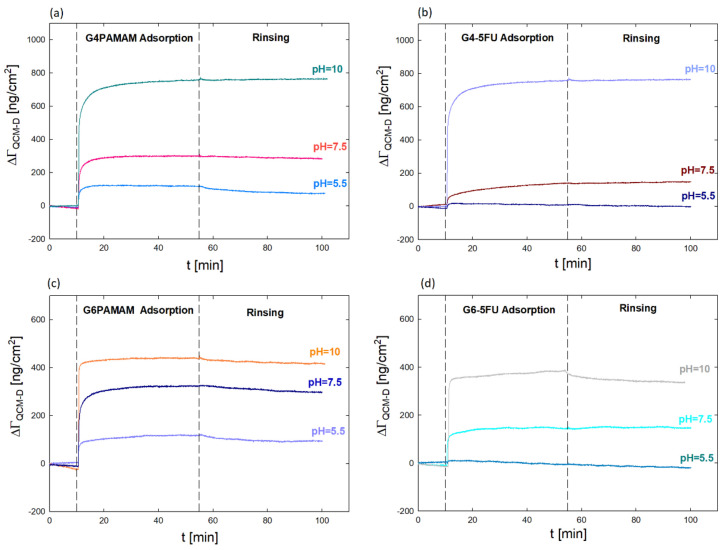
Comparison of the mass (Γ_QCM-D_) adsorbed on Au sensor surface using the QCM-D method for (**a**) G4PAMAM; (**b**) G4-5FU complexes after dialysis; (**c**) G6PAMAM (**d**) G6-5FU complex after dialysis on pH.

The quartz microbalance method with monitored energy dissipation was used to evaluate the surface properties of the obtained PAMAM dendrimer complexes with an active substance. Figure 6 shows the mass of dendrimers and G4PAMAM–5FU complexes adsorbed on the Au sensor surface using the QCM-D method.

In this case, the QCM-D method was used to determine the change in the effective charge of the dendrimer molecules after drug immobilization and its effect on the efficiency of interaction with a negatively charged surface. The analysis of the nanocarrier/negatively charged surface interaction is intended to determine the potential behavior of the dendrimer concerning negatively charged biological membranes present in the body. In addition, the determination of the mass of complexes (*Γ_QCM-D_*) adsorbed on the gold surface enabled the indication of a number of molecules of both the dendrimer (N_PAMAM)_ and the approximate number of 5FU molecules in its structure (*N_FU_*) (Table 2 and Table 3).

The adsorption process was carried out for various pH values (5.5, 7.5, and 10). The adsorbed mass was calculated using the Sauerbrey model, which assumes a proportional relationship between changes in the resonant frequency of the sensor and the mass adsorbed on the *Γ_QCM-D_* sensor [38,39]:

(4) $$\Gamma_{QCM-D}=-C\;\frac{\Delta f}{n}$$

where Δ*f* changes in the resonance frequency (Hz), C—constant characteristic for quartz crystals (C = 17.7 ng/cm^2^), *n*—number of overtones [38].

Based on QCM-D measurements, it was verified that dendrimer molecules’ adsorption efficiency is higher than that of complexes on the gold surface. The value of the adsorbed mass increases with the pH value. The most increased mass was obtained for solutions at pH 10, where the dendrimer has an isoelectric point, and the lowest at pH 5.5, when the dendrimer molecules are strongly positively charged. These studies confirm the close relationship between the degree of protonation of functional groups of the dendrimer molecule and the effectiveness of adsorption and the role of electrostatic and hydrophobic interactions [22,26,27,40].

The adsorbed mass for G4PAMAM is twice as large as for G6PAMAM, which gives almost four times more adsorbed particles per cm^2^ at pH = 5.5 and 7.5 and nearly eight times more particles/cm^2^ at pH = 10 (Table 3). The adsorbed molecules are twice lower in the complexes than in the original dendrimer under the same conditions (Table 4). The difference in the G4-5FU and G6-5FU complexes’ behavior compared to the original dendrimers confirms the changes in the dendrimer molecule’s effective charge resulting from the immobilization of drug molecules on its surface. In the case of the G6-5FU complex at pH = 5.5, the adsorption efficiency is negligible and tends to desorb.

### 2.3. Cytotoxicity Studies

**Figure 7:**
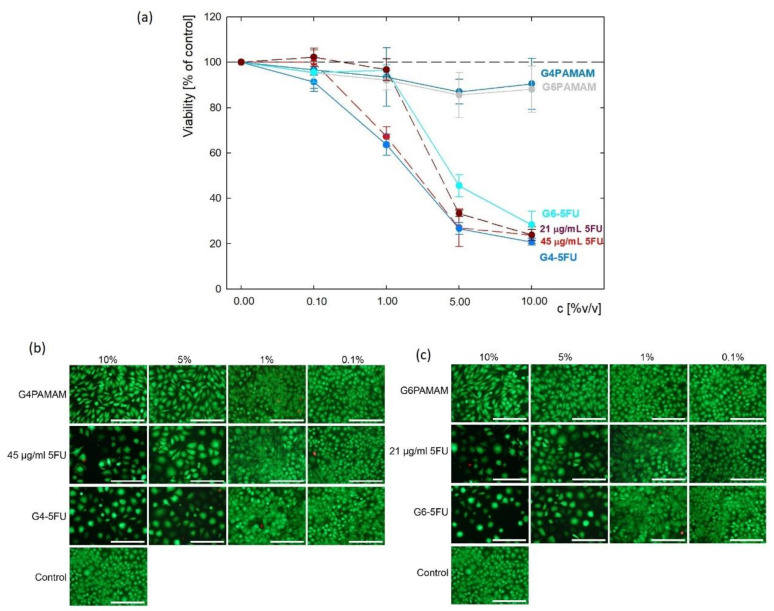
The initial evaluation of cytotoxicity of dendrimers and their complexes with 5FU against L929 mouse fibroblasts: (**a**) cell viability determined via metabolic activity assay and live/dead fluorescence microscopy images of the cells cultured for 7 days in presence of (**b**) G4PAMAM dendrimers, G4-5FU complexes and 5FU with concentration corresponding to its concentration in complex; (**c**) G6PAMAM dendrimers, G6-5FU complexes and 5FU with concentration corresponding to its concentration in complex (scale bar: 200 µm).

PAMAM dendrimers can enter the cells by endocytosis, and their configuration depends on the pH of the surrounding [41]. Albertazzi et al. demonstrated that dendrimers can be internalized by both clathrin-dependent endocytosis and micropinocytosis in the case of HeLa cells [42]. L929 fibroblasts were used as a model cell line in accordance with ISO 10993-5 standard (tests for in vitro cytotoxicity). Initial evaluation of cytotoxicity of dendrimers and their complexes with 5FU was performed using these cells. The viability of the cells (Figure 7a) cultured in the presence of G4PAMAM and G6PAMAM unloaded dendrimers was above 80% compared to untreated cells (control) (e.g., G4PAMAM: 83.0 ± 4.7%, G6PAMAM: 88.1 ± 3.8% of control on day 7). No significant difference between these two types of dendrimers was found. The cells’ decrease in viability after the exposure to 5FU alone was observed. The initial concentration of pure 5FU corresponded to its concentration in the complexes and was 21 μg/mL and 45 μg/mL. For low drug content (0.1% *v*/*v*), no differences in the effect of 5FU on cell metabolic activity were observed. Visible changes started to appear at 1% *v*/*v* 5FU content and the viability dropped to 67.3 ± 4.3% for an initial concentration of 45 μg/mL, while at the lower concentration the viability remained at 96.7 ± 4.8%. For higher concentrations of 5% *v/v*, slight differences in 5FU activity are observed, while at 10% *v*/*v* content both drug concentrations are no longer changed and the viability remains at about 24%. These results show that increasing the concentration of 5-fluorouracil above 2.1 μg/mL does not significantly affect fibroblast viability.

Similar effects were observed for both 5FU-loaded complexes. After 7 days of incubation, the cell culture’s viability in the presence of G4PAMAM–5FU and G6PAMAM–5FU complexes were 20.8 ± 1.9% and 28.3 ± 2.2% of control, respectively.

Cell viability decreased significantly upon the addition of 1% *v/v* G4-5FU and 5% *v*/*v* G6-5FU corresponding to pure 5FU solution. Besides viability evaluation using metabolic activity assay, fluorescent live/dead staining was performed to observe cell morphology and confirm previous results (Figure 7b,c, Tables S1 and S2). In all samples, most observed cells were viable, with less than 2% of dead cells. However, in the case of 5FU, G4PAMAM–5FU, and G6PAMAM–5FU treated cells, the number of cells was significantly decreased as compared to control cells and the cells treated with unloaded dendrimers. Those cells’ morphology differed from untreated cells, as the cells were enlarged and several apoptotic bodies can be observed. This observation aligns with the mechanism of action of 5FU being an antimetabolite and an inhibitor of thymidylate synthase (TS). Decreased activity of TS blocks the synthesis of a nucleotide—pyrimidine thymidylate—necessary for DNA replication, thus leading to reduced cell proliferation [43,44]. Regarding the cells incubated in the presence of unloaded dendrimers, their number was visibly decreased compared to control cells, but their morphology was similar to untreated cells. Good biocompatibility of unconjugated PAMAM dendrimers was also demonstrated by other authors, who synthetized G4 PAMAM dendrimers, no cytotoxicity against L929 cells was observed below the concentration of 50 µg/mL [45]. As indicated by Vu et al. PAMAM-based dendrimers can be cytocompatible with L929 even at 500 µg/mL [46].

**Figure 8:**
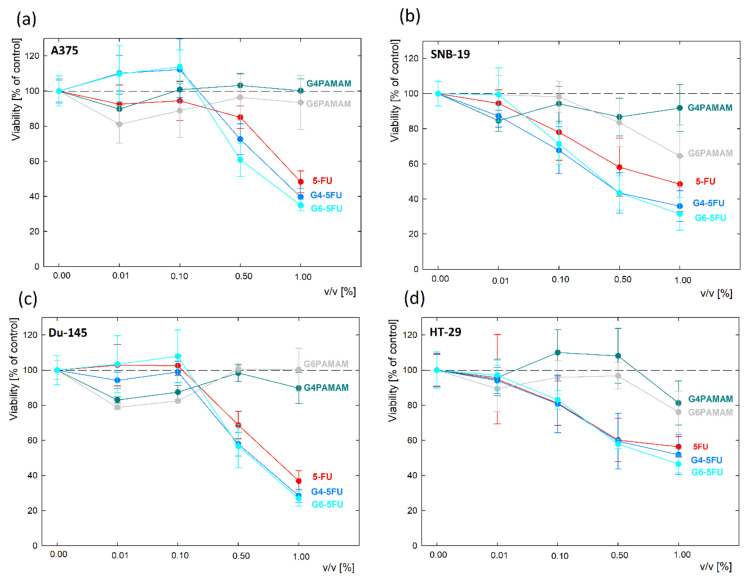
The cytotoxicity of G4PAMAM and G6PAMAM dendrimers and their complexes with 5FU towards cell lines of: (**a**) melanoma (A375); (**b**) glioblastoma (SNB-19); (**c**) prostate cancer (Du-145); (**d**) colon adenocarcinoma (HT-29), measured with MTS assay following a 7 day incubation with the compounds.

After the initial assessment of cytotoxicity against L929 cell line in an analogous manner, the viability of four malignant cell lines was estimated. Based on preliminary measurements on a normal cell line, the concentration of components was optimized. Solutions of both pure dendrimers and complexes were diluted against a maximum initial 5-fluorouracil content (45 μg/mL) ranging from 0.01–1% *v*/*v*. The viability assay demonstrated various cell responses to unconjugated dendrimers depending on the origin of a cell line; however, all four cancer cell lines showed decreased viability after treatment with 5FU, and the drug’s effect was slightly enhanced by conjugation with PAMAM dendrimers (Figure 8, Tables S3–S6). Unconjugated G4PAMAM dendrimers presented no significant toxicity towards A375, SNB-19, and Du-145 cell lines and caused a slight decrease in HT-29 cells’ viability after 7 days of incubation by less than 20% (*p* = 0.0088). G6PAMAM dendrimers appeared to be slightly more toxic; however, they did not cause a drop in cell viability by more than 30% in either of the tested cell lines. Whilst melanoma and prostate cancer cells were negatively affected only by a short incubation with a G6PAMAM dendrimer solution (up to three days), the cell lines from glioblastoma and colon carcinoma initially showed no drop in viability but their proliferation was significantly hindered after seven days of incubation with the tested compound. All of the cell lines showed a significant decrease in proliferation in time following incubation with 5FU, G4PAMAM–5FU and G6PAMAM–5FU (*p* < 0.05). In melanoma cells, conjugation with a dendrimer evoked a similar increase in drug toxicity after 7 days of incubation, regardless of the dendrimer’s generation (*p* < 0.01). None of the tested cell lines presented statistically significant differences between the viability of cells incubated with G4PAMAM-FU or G6PAMAM–5FU. SNB-19 cells showed a similar response to conjugated and unconjugated 5FU and statistically significant differences between drug and conjugate were detected only after 7 days of incubation with G6PAMAM–5FU (*p* = 0.0097), causing a decrease in viability to 32% (compared to 48% in cells incubated with 5FU). For both Du-145 and HT-29 cell lines, we observed a slight enhancement in the drug’s toxicity after conjugation with dendrimers; however, the difference did not reach statistical significance.

To broaden the analysis of anticancer activity, for all of the tested cancer cell lines, we calculated IC50 dose for unconjugated 5-FU and G4PAMAM/G6PAMAM–drug conjugates (Table 4). The maximum tested concentration of dendrimers alone (G4PAMAM and G6PAMAM) was 25 µg/mL in the following study. It corresponded to 4.5 µg/mL of 5-FU in G4PAMAM–5FU, which was sufficient to effectively suppress the proliferation of both normal and malignant cell lines.

In order to examine if any of the tested compounds is more selective towards cancer cell lines compared to normal fibroblasts, we calculated the selectivity index (SI). SI is a pure IC50 value ratio in non-cancer cell line (in our study L929) to IC50 value in cancer cell line (A375, SNB-19, Du-145, HT-19). Both IC50 and SI values are presented in Table 4. SI values exceeding 1.0 indicate the compounds of considerable anticancer specificity [47]. Our study demonstrates poor selectivity of 5-fluorouracil towards cancer cells; however, the drug’s selectivity was enhanced by the conjugation with dendrimers. The most favorable effect was obtained for G6PAMAM–5FU, increasing 5FU selectivity by over 66% in A375 and SNB-19 cells. Merging drug with G4PAMAM dendrimer increased in its selectivity by up to 36% (SNB-19 cells) (Table 5).

5-FU exerts its anticancer activity by inhibiting thymidylate synthase (TS) and incorporating its metabolites into RNA and DNA [14]. Primary or secondary resistance to 5-FU is a common phenomenon in cancer treatment. Resistance to fluoropyrimidines has been discovered to be a multifactorial event, including cell metabolism, molecular mechanisms, oxidative stress, protection from apoptosis, resistance via cell cycle kinetics and hindered drug transportation [48,49]. No chemotherapeutic drug can act efficiently if the appropriate concentration is not reached in target cells. More than 80% of the administered 5-FU dose is eliminated by the dihydropyrimidine dehydrogenase catabolism [50].

Consequently, 5-FU delivery to the tumor cells may be a severe problem in the treatment of cancer [51]. That type of resistance to 5-FU could be overcome through a better control of its intratumoral activation and the use of an encapsulated formulation [49]. Conjugation with for example dendrimers may provide increased drug stability. It should also prolong circulation of the conjugates in the bloodstream, thus enabling more drug molecules to reach their site of action—the tumor—via the enhanced permeability and retention effects [46].

## 3. Materials and Methods

### 3.1. Materials

A solution of 5-fluorouracil (5FU) was purchased from Sigma-Aldrich (St. Louis, MO, USA). The carriers used were poly(amidoamine) dendrimers (PAMAM) 4.0 and 6.0 generation (Dendritech, Inc. Michigan; Midland, MI, USA). All solutions were prepared in deionized water, adjusting the pH using sodium hydroxide (NaOH) and hydrochloric acid (HCl) solutions.

### 3.2. Dynamic Light Scattering (DLS)

The carrier particle size was determined using the dynamic light scattering method using a Malvern Nano ZS analyzer. The measurement was performed for both generations of dendrimers with a concentration of 70 µM and 17 µM for G4PAMAM and G6PAMAM, respectively.

### 3.3. Laser Doppler Velocimetry (LDV)

The electrophoretic mobility (µe) was determined using a Malvern Nano ZS analyzer. Measurements were made in the pH range of 2–11 for aqueous solutions of PAMAM–5FU complexes and dendrimers with concentrations of 70 µM (G4PAMAM) and 17 µM (G6PAMAM). Measurements of the complexes’ electrophoretic mobility were carried out after dialysis for dendrimer–drug molar ratios of 1:75 and 1:100.

### 3.4. Preparation of PAMAM–5FU Complexes

PAMAM–drug complexes were formed as aqueous solutions for dendrimer concentrations of 17.6 µM (G4PAMAM) and 4.3 µM (G6PAMAM). The dendrimer concentration was constant, and the carrier–drug molar ratio was 1:800. During the formation of the complexes, the variable parameter was the pH, which was 5.5, 7.5, and 10. The PAMAM–5FU complexes were mixed for 24h in the darkness at room temperature (298 K).

### 3.5. Dialysis of Complexes

The complexes were performed using a dialysis membrane to remove drug molecules that were not bound to the carriers (MWCO 3.5 kDa), ThermoFisher (Waltham, MA, USA). The dialysis process was carried out in distilled water at an appropriate pH. Dialysis was performed for 24 h at room temperature (298 K).

### 3.6. UV-Vis Spectrophotometry

The Thermo Scientific Evolution 201 UV-Vis spectrophotometer was used to determine the concentration of 5FU bound to the dendrimer. The UV-Vis spectra were measured in the wavelength range of 190–500 nm.

### 3.7. Quartz Microbalance with Energy Dissipation (QCM-D)

The adsorption process of dendrimers and PAMAM–5FU complexes on the gold surface was carried out using the Q-Sense E1 device. The concentration of G4PAMAM and G6PAMAM dendrimers were 17.6 µM and 4.3 µM, respectively. Adsorption was carried out for three pH values of the solutions (5.5, 7.5, and 10). The mass adsorbed on the sensor surface was calculated based on the Sauerbrey model.

### 3.8. Nuclear Magnetic Resonance (NMR) Spectroscopy

H^1^ NMR spectra of G4PAMAM dendrimers and the G4-5FU complex were obtained using a Bruker Advance 600 MHz spectroscope (T = 298 K). The measurement was performed for a dendrimer solution with a concentration of 140 µM at pH = 10. Both G4PAMAM and G4-5FU solutions were prepared in D_2_O.

### 3.9. Cytotoxicity against L929 Cell Line

Initial evaluation of dendrimers and PAMAM–5FU complexes was performed using mouse fibroblasts L929 (European Collection of Cell Cultures, Salisbury, UK) cultured in DMEM (Dulbecco’s Modified Eagle Medium, PAN BIOTECH, Aidebach, Germany) supplemented with 10% fetal bovine serum (FBS, Biowest, France) and 1% penicillin/streptomycin (PAA, Linz, Austria). Cell culture was carried out in sterile conditions at 37 °C, 5% CO_2_, and increased humidity. The cells were seeded in transparent 96-well plates at 5000 cells per well in 100 µL of complete cell culture medium and allowed to adhere and proliferate overnight. On the second day of culture, the cell culture medium was withdrawn from the wells and replaced with 200 µL of fresh cell culture medium containing 0.1, 1.0, 5.0 and 10% *v*/*v* dendrimer or complexes solutions. The initial concentration of G4PAMAM and G6PAMAM dendrimers was 250 μg/mL. 5-FU concentration in complexes depended on their effectiveness and it was 45 μg/mL for G4-5FU complex and 21 μg/mL for G6-5FU complex. Initial drug concentration was equal to its content in complexes and it was 21 and 45 μg/mL. Cell viability was evaluated using metabolic activity assay and fluorescence live/dead staining after 7 days of incubation. The metabolic activity was assessed by resazurin reduction using AlamarBlue reagent (In Vitro Toxicology Assay Kit, Resazurin based, Sigma Aldrich, St. Louis, MO, USA). Cell culture medium containing dendrimers or complexes was withdrawn and replaced with 150 µL fresh DMEM with 5% *v*/*v* addition of AlamarBlue reagent. After 3 h of incubation, 100 µL of medium containing AlamarBlue reagent was transferred to a black 96-well plate. The fluorescence intensity was measured at λ_ex_ = 530 nm, λ_em_ = 590 nm using a microplate reader (FluoroSTAR Omega, BMG Labtech, Ortenberg, Germany). The percentage of resazurin reduction was calculated according to the following formula:

(5) $$Resazurin\;reduction\;(\%)=\frac{F_{s}-F_{0\%}}{F_{100\%}-F_{0\%}}*100\%$$

where: *F_s_*—fluorescence intensity for the sample, *F_0%_*—fluorescence intensity of cell culture medium containing non-reduced AlamarBlue reagent, *F_100%_*—fluorescence intensity of cell culture medium containing completely reduced AlamarBlue reagent (autoclaved at 120 °C for 15 min). The results were presented as mean ± standard deviation for n = 3. For easier comparison, the values were recalculated as a percentage of control.

For live/dead fluorescence staining, a cell culture medium containing dendrimers or complexes was withdrawn and replaced with 100 µL of phosphate-buffered saline (PBS, PAN Biotech, Germany) containing 0.1% calcein-AM (Sigma Aldrich) and 0.1% propidium iodide (Sigma Aldrich). After 20 min of incubation, microscopic pictures were taken using Zeiss Axiovert 40 microscope with an HXP 120 °C metal halide illuminator (Carl Zeiss, Jena, Germany).

### 3.10. Efficacy of the Systems against Malignant Cells

Tests were performed on four human cancer cell lines: malignant melanoma (A375), glioblastoma (SNB-19), prostate cancer (Du-145), and colon adenocarcinoma (HT-29). Cells were maintained at 37 °C and 5% CO_2_, in a Dulbecco’s Modified Eagle’s Medium (Sigma-Aldrich) supplemented with 10% fetal bovine serum (Atlanta Biologicals, Norcross, GA), and antibiotics: 100 IU/mL penicillin, and 0.1 mg/mL streptomycin (Gibco, Gaithersburg, MD, USA). For the experiments, cells were harvested from culture flasks (Sarstedt, Warsaw, Poland) with a 0.25% trypsin-EDTA solution (Sigma-Aldrich). 200 µL of medium with suspended cells was placed to each well of a 96-well plate (Sarstedt) at cell density 1.4 × 10^3^/mL. After seeding, cells were maintained for 24 h in a CO_2_ incubator for cell attachment and homeostasis. Next, the medium from above cell culture was removed and 100 µL of 5-FU/dendrimers/conjugates diluted with a culture medium was added to wells, and plates were further kept in a CO_2_ incubator for 7 days. The initial concentrations of G4PAMAM and G6PAMAM in complexes or alone were 555 µg/mL and 1190 µg/mL, respectively. The initial concentration of 5FU alone or in complexes was 100 µg/mL. All stocks were further diluted with a culture medium to final concentrations of 1, 0.5, 0.1 and 0.01% *v*/*v*. The final concentration of 5FU conjugated with PAMAM dendrimers or unconjugated was 1 µg/mL. After 7 days of incubation cell viability was examined with the MTS assay. Shortly after the desired time of incubation, 20 µL of a CellTiter 96^®^ AQueous One Solution Reagent (Promega, Madison, WI, USA) was added to each well and then plates were placed in the incubator for another hour. Finally, following gentle mixing, the absorbance was measured at 490 nm with the GloMax^®^ Discover Microplate Reader (Promega, Madison, WI, USA). Data presented on the plots are blank-subtracted and normalized to the non-treated control. Experiments were performed in triplicates. One-way ANOVA followed by Dunnett’s multiple comparisons test was performed with the GraphPad Prism software, version 9.0. (GraphPad Software, San Diego, CA, USA) with a 95% confidence (*p* < 0.05).

## 4. Conclusions

Experimental studies show that analysis of physicochemical properties of both PAMAM dendrimers and 5-fluorouracil play a significant role in the formation of high-efficiency PAMAM–5FU complex. The effectiveness of the ligand’s binding to the dendrimers’ structure is strictly dependent on the complex formation conditions: molar ratio, ionic strength, pH, and dendrimer generation. The fact that drug molecules bind most effectively under alkaline conditions when the dendrimer is close to the isoelectric point indicates a greater influence of the ligand charge which occurs in a deprotonated form. Studies have confirmed the system’s ability to attach approximately 20 5FU molecules per dendrimer molecule for the fourth generation dendrimer and about 25 molecules for the sixth generation dendrimer. Comparing these values with the nominal number of amine groups present in the dendrimer structure, a system efficiency of 16% for G4PAMAM and 5% for G6PAMAM dendrimers was obtained.

The decrease in the zeta potential of the PAMAM–5FU systems compared to the dendrimer itself indicates a change in the carrier’s surface charge by drug immobilization. In addition, it may reveal the presence of ligand molecules on the PAMAM surface. H^1^ NMR spectra indicate the presence of drug molecules both inside the structure and on its surface. The research confirms the possibility of immobilizing the active agent in two ways and thus indicates the unique properties of the structure of dendrimers.

We demonstrated that both G4PAMAM and G6PAMAM present no toxicity towards normal cells. We also observed activity of 5-FU/PAMAM complexes in four cancer cell lines, resulting in decreasing a fluorouracil IC50 dose by up to 30%. Considering that most of the traditionally administered 5-FU is decomposed to inactive metabolites before reaching its target, drug conjugation with dendrimers seems to be a promising approach allowing us to increase both drug toxicity and stability, ultimately leading to overcoming of transportation-related drug resistance. However, the stability of 5-fluorouracil was not the subject of the present study and the issue of the conjugates’ fate in living organisms requires further investigation.

## Figures and Tables

**Table 1:** Zeta potential (ζ), effective charge (N_c_) and effective degree of ionization (α_e_) for aqueous solutions of G4PAMAM (c = 70 µM) and G6PAMAM (c = 17 µM) depending on pH.

pH	G4PAMAM			G6PAMAM		
	*ζ* [mV]	*N_c_* [e]	*α_e_* [%]	*ζ* [mV]	*N_c_* [e]	*α_e_* [%]
5.5	80.5	10.8	0.17	74.4	15.0	0.06
7.5	73.5	10.4	0.16	68.0	13.7	0.06
10	−14.0	−1.9	0.03	−4.9	−1.0	0.004

**Table 2:** Concentrations of 5FU in the most effective complexes (pH = 10) after dialysis depending on the method and dendrimer generation.

Type of Analysis (Method)	Initial PAMAM/5FU Molar Ratio	Dendrimer Generation	*c_5FU_* [μM]
Effectiveness of complex formation (UV-Vis spectrophotometry)	1:800	G4 PAMAM	462
		G6 PAMAM	143
ζ (LDV)	1:75	G4 PAMAM	138
		G6 PAMAM	44
	1:100	G4 PAMAM	185
		G6 PAMAM	58

**Table 3:** QCM-D results for G4PAMAM (c = 17.6 µM) and G6PAMAM (c = 4.3 µM) (d = 1.23 g/cm^3^).

Sample	pH	*Γ_QCM-D_* [ng/cm^2^]	*h* [nm]	*N_PAMAM_* [1 × 10^12^/cm^2^]
G4PAMAM	5.5	76 ± 5	0.65	3.2
	7.5	292 ± 4	2.37	12
	10	761 ± 3	6.19	32
G6PAMAM	5.5	97 ± 5	0.79	1.0
	7.5	308 ± 7	2.50	3.2
	10	422 ± 4	3.43	4.4

**Table 4:** QCM-D results of PAMAM–5FU complexes after dialysis (carrier–drug molar ratio 1:50, d = 1.23 g/cm^3^).

Sample	pH	*c_5FU_* [ppm]	*N_5FU_*	*Γ_QCM-D_* [ng/cm^2^]	*h* [nm]	*N_PAMAM–5FU_* [1 × 10^12^/cm^2^]
G4-5FU	5.5	4.2	1.8	3.4 ± 1	0.03	0.1
	7.5	4.6	2.0	145 ± 3	1.17	6.1
	10	13.4	5.9	348 ± 3	2.83	15
G6-5FU	5.5	1.6	2.9	-	-	-
	7.5	1.6	2.9	149 ± 2	1.21	1.5
	10	3.8	6.8	343 ± 4	2.79	3.5

**Table 5:** The half maximal inhibitory concentration (IC50) values and the selectivity index (SI) values of 5-fluorouracil alone (5-FU) or conjugated with G4PAMAM or G6PAMAM dendrimers (G4PAMAM–5FU and G6PAMAM–5FU, respectively) in 5 cell lines: mouse fibroblasts (L929) malignant melanoma (A375), glioblastoma (SNB-19), prostate cancer (Du-145) and colon adenocarcinoma (HT-29) following a 7 day incubation with the compounds.

Cell Line	L929	A375	SNB-19	Du-145	HT-29
Compound IC50 [µg/mL]					
5-FU	0.67	1.06	0.86	0.80	1.04
G4PAMAM–5FU	0.66	0.85	0.62	0.68	0.92
G6PAMAM–5FU	0.8	0.76	0.61	0.68	0.83
Compound SI					
5-FU	-	0.63	0.78	0.84	0.64
G4PAMAM–5FU	-	0.78	1.06	0.97	0.72
G6PAMAM–5FU	-	1.05	1.31	1.18	0.96

## Data Availability

Not applicable.

## Supplementary Materials

The following are available online at https://www.mdpi.com/article/10.3390/ijms222011167/s1.

## Abbreviations

PAMAM dendrimers	poly(amidoamine) dendrimers
G4PAMAM	poly(amidoamine) dendrimer of 4th generation
G6PAMAM	poly(amidoamine) dendrimers of 6th generation
5FU	5-Fluorouracil
G4PAMAM–5FU	5-Fluorouracil complex with 4th generation of dendrimer
G6PAMAM–5FU	5-Fluorouracil complex with 6th generation of dendrimer
QCM-D	Quartz Crystal Microbalance with Dissipation Monitoring
Γ_QCM-D_	Adsorbed mass measured by Quartz Crystal Microbalance with Dissipation Monitoring
h	thickness of the adsorbed layer [nm]
N_PAMAM_	number of adsorbed PAMAM molecules per 1 cm^2^
N_5FU_	number of 5FU molecules attached to one dendrimer molecule
N_PAMAM–5FU_	number of adsorbed PAMAM–5FU complex molecules per 1 cm^2^
