# Supplementary material for: Poly(amidoamine) Dendrimers as Nanocarriers for 5-Fluorouracil: Effectiveness of Complex Formation and Cytotoxicity Studies
Source: Int J Mol Sci. 2021 Oct 16;22(20):11167. doi: 10.3390/ijms222011167 (PMC8537672; doi:10.3390/ijms222011167)
Supplement: Supplementary file 1 [file ijms-22-11167-s001.zip › ijms-1390616-supplementary.pdf]

Supplementary Material

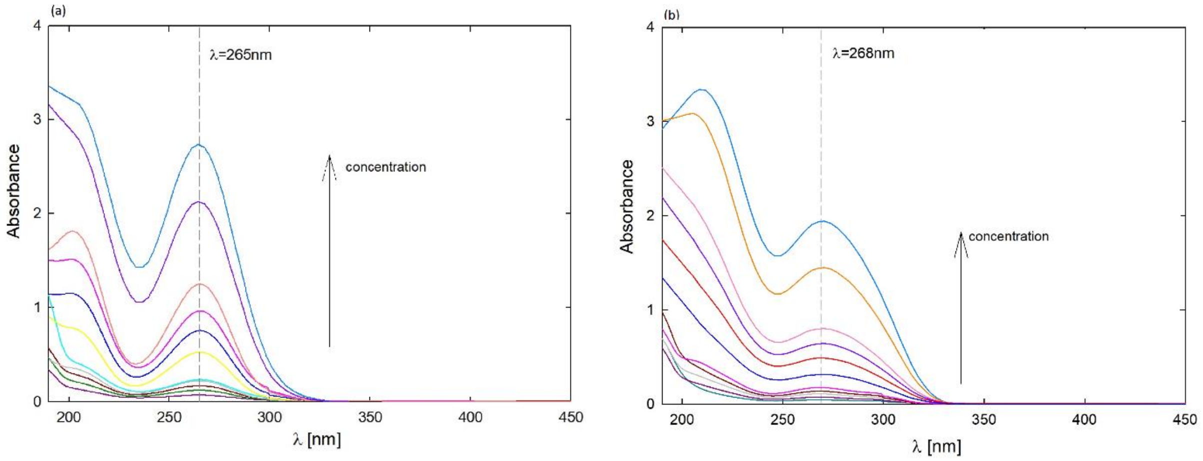

Figure S1. UV-Vis spectra of an aqueous 5FU solution (c = 7.7-192 μM). a) at pH = 7.5; b) at pH = 10.

Table S1. Statistically significant differences in L929 cell viability after incubation for 7 days in presence of 10% v/v G4PAMAM and G6PAMAM dendrimers, 5-FU and G4-5FU and G6-5FU complexes determined by one-way ANOVA followed by post-hoc Tukey test at α=0.05.

	**Control**	**G4PAMAM**	**G6PAMAM**	**5FU**	**G4-5FU**	**G6-5FU**
Control		n.s.[1]	n.s.	<0.001	<0.001	<0.001
G4PAMAM	n.s.		n.s.	<0.001	<0.001	<0.001
G6PAMAM	n.s.	n.s.		<0.001	<0.001	<0.001
5FU	<0.001	<0.001	<0.001		n.s.	n.s.
G4-5FU	<0.001	<0.001	<0.001	n.s.		0.045
G6-5FU	<0.001	<0.001	<0.001	n.s.	0.045	

[1]n.s. – not significant.

Table S2. Statistically significant differences in L929 cell viability after incubation for 7 days in presence of 5% v/v G4PAMAM and G6PAMAM dendrimers, 5-FU and G4-5FU and G6-5FU complexes determined by one-way ANOVA followed by post-hoc Tukey test at α=0.05.

	**Control**	**G4PAMAM**	**G6PAMAM**	**5FU**	**G4-5FU**	**G6-5FU**
Control		n.s.	n.s.	<0.001	<0.001	<0.001
G4PAMAM	n.s.		n.s.	<0.001	<0.001	<0.001
G6PAMAM	n.s.	n.s.		<0.001	<0.001	<0.001
5FU	<0.001	<0.001	<0.001		n.s.	0.022
G4-5FU	<0.001	<0.001	<0.001	n.s.		0.027
G6-5FU	<0.001	<0.001	<0.001	0.022	0.027	

Table S3. Statistically significant differences in A375 cell viability after incubation for 7 days in presence of 1% v/v G4PAMAM and G6PAMAM dendrimers, 5-FU and G4-5FU and G6-5FU complexes determined by one-way ANOVA followed by post-hoc Tukey test at α=0.05.

	**Control**	**G4PAMAM**	**G6PAMAM**	**5FU**	**G4-5FU**	**G6-5FU**
**Control**		n.s.	n.s.	n.s.	n.s.	n.s.
**G4PAMAM**	n.s.		n.s.	n.s.	n.s.	n.s.
**G6PAMAM**	n.s.	n.s.		n.s.	n.s.	n.s.
**5FU**	n.s.	n.s.	n.s.		n.s.	n.s.
**G4-5FU**	n.s.	n.s.	n.s.	n.s.		0.033

G6-5FU	n.s.	n.s.	n.s.	n.s.	0.033	

Table S4. Statistically significant differences in SNB-19 cell viability after incubation for 7 days in presence of 1% v/v G4PAMAM and G6PAMAM dendrimers, 5-FU and G4-5FU and G6-5FU complexes determined by one-way ANOVA followed by post-hoc Tukey test at α=0.05.

	Control	G4PAMAM	G6PAMAM	5FU	G4-5FU	G6-5FU
**Control**		n.s.	<0.001	<0.001	<0.001	<0.001
**G4PAMAM**	n.s.		<0.001	<0.001	<0.001	<0.001
**G6PAMAM**	<0.001	<0.001		0.0178	<0.001	<0.001
**5FU**	<0.001	<0.001	0.0178		n.s.	0.0194
**G4-5FU**	<0.001	<0.001	<0.001	n.s.		n.s.
**G6-5FU**	<0.001	<0.001	<0.001	0.0194	n.s.	

Table S5. Statistically significant differences in Du-145 cell viability after incubation for 7 days in presence of 1% v/v G4PAMAM and G6PAMAM dendrimers, 5-FU and G4-5FU and G6-5FU complexes determined by one-way ANOVA followed by post-hoc Tukey test α = 0.05.

	Control	G4PAMAM	G6PAMAM	5FU	G4-5FU	G6-5FU
**Control**		n.s.	n.s.	<0.001	<0.001	<0.001
**G4PAMAM**	n.s.		n.s.	<0.001	<0.001	<0.001
**G6PAMAM**	n.s.	n.s.		<0.001	<0.001	<0.001
**5FU**	<0.001	<0.001	<0.001		n.s.	n.s.
**G4-5FU**	<0.001	<0.001	<0.001	n.s.		n.s.
**G6-5FU**	<0.001	<0.001	<0.001	n.s.	n.s.	

Table S6. Statistically significant differences in HT-29 cell viability after incubation for 7 days in presence of 1% v/v G4PAMAM and G6PAMAM dendrimers, 5-FU and G4-5FU and G6-5FU complexes determined by one-way ANOVA followed by post-hoc Tukey test at α=0.05.

	Control	G4PAMAM	G6PAMAM	5FU	G4-5FU	G6-5FU
**Control**		n.s.	<0.001	<0.001	<0.001	<0.001
**G4PAMAM**	n.s.		n.s.	0.0054	0.0020	<0.001
**G6PAMAM**	<0.001	n.s.		0.0195	0.0073	<0.001
**5FU**	<0.001	0.0054	0.0195		n.s.	n.s.
**G4-5FU**	<0.001	0.0020	0.0073	n.s.		n.s.
**G6-5FU**	<0.001	<0.001	<0.001	n.s.	n.s.	
